# Nicotine Pouch Use Among US Military Personnel

**DOI:** 10.1001/jamanetworkopen.2024.51517

**Published:** 2024-12-17

**Authors:** Melissa A. Little, Kathryn M. Polaskey, Asal Pilehvari, Rebecca A. Krukowski, Kurt M. Ribisl, Teresa D. Pearce

**Affiliations:** 1 Department of Public Health Sciences, University of Virginia School of Medicine, University of Virginia, Charlottesville; 2 University of Virginia Cancer Center, University of Virginia, Charlottesville; 3 Fort Liberty Department of Public Health, Fort Liberty, North Carolina; 4 Department of Health Behavior, Gillings School of Global Public Health, University of North Carolina at Chapel Hill; 5 Lineberger Comprehensive Cancer Center, University of North Carolina at Chapel Hill

## Abstract

This cross-sectional study evaluates the prevalence of and factors associated with use of nicotine pouches among active US military personnel.

## Introduction

Military personnel use tobacco at higher rates than the general population and experience more tobacco-related disparities, particularly after transitioning back into civilian life. For example, 9.4% of military personnel use smokeless tobacco compared with 3.5% of civilians.^1^ However, little is known regarding use of nicotine pouches (microfiber sachets prefilled with dissolvable nicotine salt powder).^2^ While only 2.9% of adults reported lifetime use of nicotine pouches in 2022,^3^ US sales of these products increased 641% between 2019 and 2022,^2^ suggesting a need to investigate whether subgroups of the population are driving these sales. The current study explored nicotine pouch use among active-duty personnel to inform intervention efforts.

## Methods

The Department of Public Health at Fort Liberty, the largest installation (by population) in the US military, conducts annual tobacco and nicotine use surveillance on post. Soldiers visiting a predeployment site during the fall of 2022 (411 completed surveys) and 2023 (1546 completed surveys) were approached to complete an anonymous survey by public health employees as part of public health surveillance. Following data collection, the survey was approved by the University of Virginia institutional review board as secondary data analysis for the purposes of data analysis and publication. This study follows the Strengthening the Reporting of Observational Studies in Epidemiology (STROBE) reporting guideline for cross-sectional studies.

The assessment measured self-reported demographic characteristics and current nicotine and tobacco use. Multivariable logistic regression analyses tested the association of soldiers’ characteristics with nicotine pouch use. Analyses were conducted in Stata version 18 (StataCorp).

## Results

Of 1957 participants, most were male, young, White, single, and tobacco users (Table 1); 465 (23.8%) reported past 30-day nicotine pouch use (Table 2), of whom 93 (20.0%) reported no other past 30-day tobacco use. Use increased between 2022 and 2023 (from 20.2% to 24.7%). Users were more likely to be younger, male, White, unmarried, and report other tobacco and nicotine product use (ie, cigarettes, cigars or cigarillos, dip or smokeless tobacco, hookah, vape or e-cigarettes). In the logistic regression models, compared with those aged 17 to 24 years, participants aged 30 years or older had lower odds of using nicotine pouches. Higher education (ie, >bachelor’s degree) was associated with increased nicotine pouch use compared with those with a high school degree or General Educational Development. Use of various nicotine and tobacco products in the past 30 days increased the odds of using nicotine pouches. Participants surveyed in 2023 vs 2022 had higher odds of using nicotine pouches.

**Table 1.** Sample Characteristics

Characteristic	Participants, No. (%) (N = 1957)
Sex	
Male	1807 (92.3)
Female	150 (7.7)
Age, y	
≥30	389 (20.1)
25-29	507 (26.2)
17-24	1040 (53.7)
Race^a^	
Black	310 (15.8)
Other^b^	405 (20.7)
White	1242 (63.5)
Education	
Associate’s degree or some college	600 (30.9)
Bachelor’s degree or higher	379 (19.5)
High school graduate (or GED)	963 (49.6)
Marital status	
Single, divorced, widowed	1155 (59.0)
Married	802 (41.0)
Past 30-d tobacco use^c^	
Cigarettes or roll your own	390 (19.9)
Cigars or cigarillo	227 (11.6)
Dip or smokeless tobacco	366 (18.7)
Hookah	91 (4.6)
E-cigarette use	611 (31.2)
Survey year	
2023	1546 (79.0)
2022	411 (21.0)

Abbreviation: GED, General Educational Development.

^a^ Race was self-identified by selecting all that apply among the following: American Indian or Alaska Native, Asian, Black or African American, Pacific Islander, White, or other.

^b^ Other category includes those who self-identified as American Indian or Alaska Native, Asian, Pacific Islander, or other.

^c^ Item asked, “In the past 30 days, did you use the following products?” with response options yes or no for the following products: cigarettes or roll you own, cigars or cigarillos, dip or smokeless tobacco/snus, e-cigarettes or vape, hookah, and nicotine pouches.

**Table 2.** Nicotine Pouch User Characteristics and Multivariable Logistic Regression Analysis

Characteristic	Nicotine pouch user, No./total No. (%)	Adjusted OR (95% CI)^a^
Overall	465/1957 (23.8)	NA
Sex		
Male	451/1807 (24.9)	2.29 (1.20-4.36)
Female	14/150 (9.3)	1 [Reference]
Age, y		
≥30	60/389 (15.4)	0.62 (0.41-0.94)
25-29	124/507 (24.4)	0.96 (0.70-1.32)
17-24	278/1040 (26.7)	1 [Reference]
Race^b^		
Black	25/310 (8.1)	0.24 (0.14-0.39)
Other^c^	78/405 (19.2)	0.65 (0.46-0.91)
White	362/1242 (29.1)	1 [Reference]
Education		
Associate’s degree or some college	134/600 (22.3)	1.28 (0.94-1.75)
Bachelor’s degree or higher	88/379 (23.2)	1.89 (1.29-2.75)
High school graduate (or GED)	240/963 (24.9)	1 [Reference]
Marital status		
Single, divorced, widowed	294/1155 (25.45)	1.07 (0.81-1.42)
Married	171/802 (21.3)	1 [Reference]
Past 30-d tobacco use		
Cigarettes or roll your own		
Yes	215/465 (46.2)	1.42 (1.02-1.99)
No	250/465 (53.8)	1 [Reference]
Cigars or cigarillo		
Yes	144/465 (31.0)	1.40 (0.93-2.11)
No	321/465 (69.0)	1 [Reference]
Dip or smokeless tobacco		
Yes	239/465 (51.4)	5.11 (3.74-6.97)
No	226/465 (48.6)	1 [Reference]
Hookah		
Yes	72/465 (15.5)	4.05 (1.99-8.22)
No	393/465 (84.5)	1 [Reference]
E-cigarette		
Yes	295/465 (63.4)	3.49 (2.60-4.68)
No	170/465 (36.6)	1 [Reference]
Survey year		
2023	382/465 (24.7)	1.78 (1.26-2.52)
2022	83/465 (20.2)	1 [Reference]

Abbreviations: GED, General Educational Development; NA, not applicable; OR, odds ratio.

^a^ Estimate from a fully adjusted multivariable logistic regression model that included all covariates based on theoretical relevance and previous literature. Variables were assessed for multicollinearity using variance inflation factor tests and found no significant concerns.

^b^ Race was self-identified by selecting all that apply among the following: American Indian or Alaska Native, Asian, Black or African American, Pacific Islander, White, or other.

^c^ Other category includes those who self-identified as American Indian or Alaska Native, Asian, Pacific Islander, or other.

## Discussion

Nicotine pouch use was 10-fold higher than previous estimates in the general adult population.^3^ More education was associated with higher use of nicotine pouches; previous studies have found nicotine pouches associated with both higher and lower education.^4^ These data indicate that nicotine pouch use in military populations merits additional focus in prevention and cessation efforts. Future research should examine nicotine pouch use in other subpopulations that historically report higher rates of tobacco use. Limitations include the use of a convenience sample collected as part of public health surveillance data with an unknown response rate, a cross-sectional self-report survey, and the restricted ranges of age and sex. It is possible that other tobacco product users were more likely to use nicotine pouches for harm reduction; the rates of nicotine pouch use for harm reduction in general and among this population should be explored in future research.

Given that roughly 200 000 military personnel transition back to civilian life annually, further surveillance of nicotine pouch use is needed to prevent the continuation of high nicotine and tobacco use rates into civilian life. This surveillance can also help to design and inform programs to assist military and civilian populations in cessation activities, improving overall health outcomes and easing their transition to civilian life.

## Notes

Supplement.
Data Sharing Statement
