# Supplementary material for: Nicotine Pouch Use Among US Military Personnel
Source: JAMA Netw Open. 2024 Dec 17;7(12):e2451517. doi: 10.1001/jamanetworkopen.2024.51517 (PMC11653119; doi:10.1001/jamanetworkopen.2024.51517)
Supplement: Supplement. — Data Sharing Statement [file jamanetwopen-e2451517-s001.pdf]

Data Sharing Statement

Little. Nicotine Pouch Use Among US Military Personnel. *JAMA Netw Open*. Published December 17, 2024. doi:10.1001/jamanetworkopen.2024.51517

Data
Data available: No

Additional Information
Explanation for why data not available: This data is owned by the Department of Defense and is not able to be shared.
